# Hepatitis B-Induced Hepatocellular Carcinoma: Understanding Viral Carcinogenesis and Disease Management

**DOI:** 10.3390/jcm14072505

**Published:** 2025-04-07

**Authors:** Yasamin Farbod, Husain Kankouni, Maryam Moini, Scott Fung

**Affiliations:** 1 Division of Gastroenterology and Hepatology, McGill University, Montreal, QC H3A 0G4, Canada; 2 Division of Gastroenterology and Hepatology, University of Toronto, Toronto, ON M5G 2C4, Canada; 3 Division of Gastroenterology, McMaster University, Hamilton, ON L8S 2A5, Canada; 4 Department of Medicine, University of Toronto, Toronto, ON M5G 2C4, Canada

**Keywords:** hepatitis B virus (HBV), hepatocellular carcinoma (HCC), antiviral therapy

## Abstract

Hepatitis B virus (HBV) infection is a leading cause of chronic liver disease and liver cancer worldwide. Hepatocellular carcinoma (HCC) remains one of the major causes of cancer-related mortality globally. Effective prevention and management strategies for HBV infection are crucial in reducing liver-related complications, including HCC. HBV plays a distinct role in liver carcinogenesis, and there is growing knowledge about the factors contributing to its oncogenic potential. With advancements in HCC management, special attention must be given to the treatment of HBV infection in patients with HBV-induced HCC. In this review, we summarize current insights into the carcinogenic mechanisms of HBV and discuss the latest approaches to managing HBV-induced HCC.

## 1. Introduction/Epidemiology

Chronic hepatitis B virus (HBV) infection is associated with significant mortality and morbidity worldwide. Over the past decade, there have been increasing efforts to screen for and diagnose complications of HBV such as cirrhosis and hepatocellular carcinoma (HCC). Risk factors for liver-related complications include viral genotype, elevated liver enzyme alanine transferase (ALT), HBV viral load, and coinfections, as well as comorbidities such as diabetes mellitus and metabolic syndrome [1].

The infection burden is variable across the world and depends on the dominant modes of transmission in various regions. The most common route of transmission globally remains mother-to-child transmission, particularly in southeast Asia and sub-Saharan Africa. The risk of developing chronic hepatitis B is strongly correlated with the age of transmission. Hence, infection acquired through vertical transmission amongst infants and young children leads to chronic infection and its complications in >90% of cases, whereas infection acquired through horizontal transmission such as sexual contact or injection drug use in immune-competent adults generally resolves without complications of chronic liver disease. Transmission via transfusion of blood or blood products has been largely eliminated due to careful screening of blood products [1].

## 2. Incidence and Prevalence

**Figure 1:**
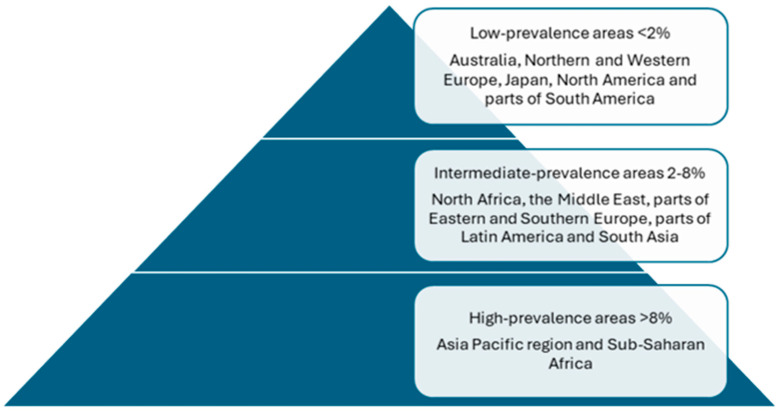
Global prevalence of hepatitis B infection.

High-prevalence HBV infection areas encompass regions where HBsAg (hepatitis B surface antigen) seroprevalence is more than 8% of population. This includes many countries in the Asia Pacific region and sub-Saharan Africa. In a recent systematic review, HBV seroprevalence remained the highest in Africa and Asia [2]. Populations with an intermediate prevalence rate are those with an HBV infection rate of 2–8%, and low-prevalence populations are those with less than a 2% HBV infection rate (Figure 1). Notable in many areas, indigenous populations have a high prevalence of chronic hepatitis B as well as an increased burden of associated liver disease and complications [1,3].

After a decade of stable rates in the United States, the rate of hepatitis B acute infection decreased in 2020 and again for the second time in 2021. The Centers for Disease Control and Prevention (CDC) estimated 13,300 infections in 2021, based on 2045 acute hepatitis cases reported from 47 states. This is interpreted as a 14% decrease in acute hepatitis B infections compared to 2020. Limiting factors such as the COVID-19 pandemic and limited access to healthcare may have contributed to such results as well. Moreover, in 2021, a total of 14,229 new cases of chronic hepatitis B (5.9 cases per 100,000 people) were reported [4]. The World Health Organization (WHO) estimates for the year 2022 showed 254 million people living with chronic hepatitis B infection, as well as 1.2 million new infections per year. Most deaths from hepatitis B are estimated to be a result of cirrhosis and hepatocellular carcinoma [5].

## 3. Screening Population

In 2023, the CDC recommended screening for HBV infection in all adults aged 18 years or older at least once in their lifetime. A triple panel test including HBsAg, anti-HBs (anti-surface antibody), and anti-HBc (anti-core antibody) should be employed regardless of risk factor disclosure [6]. Moreover, the CDC also recommended testing for HBsAg and anti-HBs in all infants born to HBsAg-positive mothers. Pregnant persons must be screened for HBV during each pregnancy, preferably in the first trimester.

Hepatitis B screening is recommended among high-risk individuals. This group includes individuals who have sexual contact with patients known to be infected with hepatitis B; immunocompromised individuals such as those living with HIV; persons who inject drugs (PWIDs); men who have sex with men (MSMs); sex workers; the carceral and institutionalized population; and persons who have received organ or blood donations prior to 1990, especially in countries with resource constraints. Routine screening is also recommended in patients with hepatitis C virus (HCV) infection. In the absence of identifiable risk factors, screening for HBV is recommended in persons with clinical and laboratory findings suggestive of chronic liver disease, acute hepatitis, HCC, or a previous diagnosis of other liver diseases [6,7,8].

## 4. Hepatitis B Vaccination and HCC

Since chronic hepatitis B is a leading cause of HCC worldwide, it is therefore essential to prevent HBV infection in order to reduce the prevalence of HCC. Several studies have supported the role of HBV vaccination in reducing the rate of HCC. In a study from Taiwan, it was shown that nationwide universal HBV vaccination (implemented in 1984) was associated with a reduced annual incidence rate of HCC in children aged 6–14 years and also a reduced mortality rate related to HCC [9]. Another cohort study in 2022 in Taiwan examined the national health database for HCC and death rates among 43,604 children and adults aged 5 to 39 years. The authors concluded that the national hepatitis B vaccination program and antiviral therapy were associated with substantial reductions in HBV-related end-stage liver disease [10]. Wang et al. have shown significant reductions in HCC-related mortality in a county in China that was the first to implement universal childhood vaccination against HBV. The reduction in mortality was observed over time and in comparison with a neighboring county that adopted the vaccination program years later [11]. In a population-based cluster randomized controlled interventional study, it was shown that neonatal HBV vaccination significantly decreased the risk of primary liver cancer and mortality from end-stage liver disease and fulminant hepatitis in young adults in rural China [12]. Universal newborn vaccination, mass screening, and immunization of susceptible Alaska Natives was shown to result in the elimination of HCC and acute HBV infection in children [13].

## 5. HBV Genotypes

There are at least 10 genotypes of HBV globally, some of which have been associated with increased risk of liver disease with some therapeutic implications. Different genotypes have variable risk of liver disease progression and responses to treatment with Interferon-alpha.

Genotypes A and B have been associated with a more favorable response to Interferon-alpha-based therapy when compared to genotypes C and D. However, it appears that all HBV genotypes respond similarly favorably to nucleos(t)ide analog (NA) therapy [14].

HBV genotypes C and D have been associated with a lower rate of spontaneous HBeAg (hepatitis B e antigen) seroconversion, as well as a higher risk of developing cirrhosis and HCC when compared to genotypes A and B [15,16,17,18]. Overall, HBV genotypes yield important prognostic information and may be useful in determining treatment response to immunomodulatory therapy. Genotype C has been shown to be associated with an increased risk of HCC as an independent risk factor [17]. In a recently published long-term follow-up study of patients with chronic HBV infection, HBV genotype, baseline HBV DNA level, and hepatic histological score were predictors of HCC. In that study, poorer outcome was reported in patients with genotypes B and C, with a higher rate of HCC and a lower rate of HBsAg loss compared with genotypes A and D [19]. It has been shown that deletion in the preS1 start codon commonly found in genotype C HBV is associated with HCC progression. PreS1 deletion activates the IRE1 pathway, is associated with hepatocyte proliferation and liver inflammation, and results in increased HBV replication and HCC development [20].

## 6. Mechanism of HBV-Induced HCC

Over the years, it has been shown that HBV infection can induce liver disease and cause disease progression, via a variety of different mechanisms, eventually leading to a malignant transformation. Chronic HBV infection can progress to advanced liver fibrosis and eventually cirrhosis as a risk factor for HCC. On the other hand, there are specific virus-related mechanisms associated with the carcinogenicity and development of HCC in chronic HBV infection even in the absence of cirrhosis. These mechanisms include HBV gene integration, causing genomic instability, and the activation of cancer-promoting signaling pathways [21]. Moreover, there is currently expanding research on the roles of epigenetics, exosomes, autophagy, and metabolic regulation and immune suppression [22]. Jiang et al. have reviewed the literature for the mechanisms of the carcinogenicity of HBV and inducing HCC [23].

HBV contains a core protein capsid and a lipid envelope. The envelope is made of a lipid bilayer with various proteins, which contains the S, pre-S1, and pre-S2 antigens, collectively known as the hepatitis B surface antigen (HBsAg). Moreover, the core particle contains the HBV core antigen (HBcAg); double-stranded-DNA; and HBV DNA polymerase, which is responsible for the replication of viral genome. The nucleocapsid-related E-antigen secreted into the serum is known as HBeAg and is thought to participate in immune tolerance or regulation of the host. The HBV genome contains four open reading frames (ORFs), ORF P/S/C/X. ORF P encodes DNA polymerase, reverse transcriptase, RNAse H, and proteins with primase activity. ORF S is responsible for the S genes, and pre-S2 and S1 proteins. ORF C encodes HBcAg and HBeAg. ORF X encodes the minimum X protein, which regulates or enhances promotors of homologous or heterologous genes, closely related to the ability of HBV to infect cells as well promote the development of HCC [23,24].

Mutation is a known mechanism that may lead to malignant transformation. Mutations both in HBV or somatic genes can result in malignant transformation. Various factors are associated with mutations including chronic viral infections, host immune stress, and other environmental factors such as aflatoxin. Mutations in G1896A (precore STOP) of the pre-C region and T1762/A1764 in the basal core promoter (BCP) have been associated with more severe liver disease and HCC in some studies. Certain mutations in pre-S1 and pre-S2 genes have also been reported in patients with chronic hepatitis B and HCC. Such mutations can lead to a reduction in HBeAg production or aberrant HBsAg production, which may result in HCC [25,26]. HBV proteins such as Hepatitis B x (HBx), HBs, and HBc can cause oxidative damage via reactive oxidative species, directly playing a role in inducing liver fibrosis and liver cancer [26,27]. HBV induces the expression of pro-oncogenic MAPK14 [28], which promotes HBV replication and tumor cell survival.

Epigenetics may result in the remodeling of HBV genes by changing the methylation states of HBV DNA and post-translationally modifying histones, known to be a key pathogenic factor for HBV-related HCC [29,30]. A meta-analysis indicated that HBV could induce DNA methylation, which could lead to the carcinogenesis and development of HCC [31]. In addition, in an analysis of viral-induced (HBV and HCV) HCC versus non-viral HCC, more hypermethylated differential methylation was observed in viral-induced HCC tissue [32].

The activation of the Wnt/beta-Catenin pathway, a tumor signaling pathway, is found in 66% of HCC cases. HBV is known to activate this pathway: HBx interacts with MyH9 to activate the Wnt/beta-Catenin/c-Jun pathway, leading to cell proliferation, metastasis, and resistance to various chemotherapeutic agents. HBx binds to APC and replaces the degradable complex for Beta-catenin, leading to the accumulation of Beta-Catenin, activating Wnt signaling, which promotes malignant transformation [33]. The PI3K/Akt pathway is involved in the control of cell growth and is abnormally activated in various tumors. PTEN is one of the genes that can negatively regulate this pathway, and HBV is known to inhibit PTEN and activate Akt, resulting in hepatocyte carcinogenesis [34,35,36,37].

In summary, various HBV mutations and several pathogenic factors are implicated in HCC development. These include the role of HBx protein, HBV epigenetics, Wnt/beta-Catenin, and PI3K/Akt pathways activation. Table 1 summarizes the main known mechanisms of HBV-induced HCC.

## 7. Role of Hepatitis Delta Virus Coinfection

Hepatitis delta virus (HDV) coinfection has been shown to increase the risk of cirrhosis and HCC development compared to HBV mono-infection [38,39]. The HDV antigen (HDAg) was previously not thought to be directly cytotoxic to hepatocytes [40]. However, further studies using adenoviral vectors carrying functional HBV and HDV genomes demonstrated that HDAg contributes to chronic liver injury [41,42]. The large HDAg (L-HDAg) in the liver and kidney may disrupt the TNF-alpha-NF-κB signaling pathway [28,40,41,42,43,44], further resulting in chronic inflammation and ultimately fibrosis and cirrhosis [45]. The activation of transforming growth factor-β (TGF-β) has been identified in the coinfection of HBV/HDV, a signaling pathway that regulates cell growth, differentiation, apoptosis, and may lead to liver fibrosis and cirrhosis [46].

## 8. Does HBV Treatment Reduce the Risk of HCC?

Several retrospective studies have documented a reduction in the risk of HCC in large cohorts of HBV patients receiving treatment with entecavir. Entecavir and tenofovir are recommended as the first-line therapies for chronic HBV infection in most treatment guidelines. These therapies are highly effective and well tolerated. Improvement in hepatic inflammation as measured by ALT or on biopsy was found to be equivalent between these two agents [47]. Studies to assess the effect of these agents on HCC development in a prospective fashion would be unethical and therefore not possible to perform [47]. However, HCC risk calculators have been used in studies of prospective randomized clinical trials, which have reported significant HCC risk reduction in both cirrhotic and non-cirrhotic patients receiving tenofovir-based NA therapy [48].

One potential strategy to reduce the risk of HCC in patients with chronic hepatitis B may be treatment with PEG-IFN-alpha, due to its immunomodulatory, anti-oncogenic, and antifibrotic effects and higher rates of HBsAg seroclearance compared to NA therapy. However, most patients opt for NA over PEG-IFN due to its convenience of administration and lack of side effects. Despite treatment with PEG-IFN, many patients have persistently detectable serum HBsAg, and HBeAg with detectable HBV DNA. As such, the risk of ongoing hepatocellular damage occurs, and cirrhosis and HCC risk remains elevated. Therefore, a reasonable goal would entail early treatment of those with active liver disease and detectable HBV DNA in order to prevent the development of cirrhosis with PEG-IFN with or without NA combination therapy. NA therapy has been shown to reduce progression to cirrhosis and risk of HCC. For example, the VIRGIL study showed that those who had a sustained response to entecavir had reduced risk of decompensation, HCC, and death compared to those who had a sustained viral response [49].

Taken together, the bulk of the evidence suggests that long-term NA therapy with or without PEG-IFN can reduce the risk of HCC in patients with chronic hepatitis B.

## 9. Treatment of HCC in HBV Patients and a Spotlight on Prophylaxis

Currently, several treatment options exist for HBV patients diagnosed with early- and intermediate-stage HCC. These include surgical resection, liver transplantation, local ablation therapies, transarterial therapies, and systemic therapies including antiangiogenic targeted therapies and immunotherapy (Table 2). The planning of treatment strategies needs multidisciplinary team involvement [50].

Liver transplantation is recommended as a first-line option for BCLC stage A HCC or stage B HCC in those who meet the extended liver transplant criteria when unsuitable for resection. In patients with early-stage HCC and compensated liver disease, surgical resection and radiofrequency ablation (RFA) are equally effective for small solitary tumors as a curative treatment. For those who are successfully treated by resection or RFA, liver transplantation may be reasonably delayed or reserved for those who show HCC recurrence, in order to allow prompt liver transplantation for those with more advanced tumors and/or decompensated cirrhosis (with medium–high model for end-stage liver disease [MELD] scores) [51].

The inhibition of HBV replication in HBV-infected patients with HCC can help improve survival and reduce the risk of HCC recurrence by improving long-term liver function. In general, patients with HCC with or without cirrhosis are recommended to remain on NA therapy lifelong. Overall, treating chronic hepatitis B infection in patients who have been treated for HCC can be challenging, but it is generally safe and well tolerated. Long-term treatment reduces liver inflammation and hepatic fibrosis, which decreases the risk of hepatic decompensation and tumor recurrence. In such cases, treatment with NA is much preferred over IFN therapy, which has a potential risk of liver decompensation in advanced cirrhosis. In a study using lamivudine in post-resection patients with HCC, a reduction in the risk of tumor recurrence was seen in 40.6% of lamivudine patients vs. 49.7% in the untreated population, while mortality was reduced to 24.6% vs. 36.4%, respectively [52].

Transarterial chemoembolization (TACE) has been reported to be associated with an increased risk of HBV reactivation and hepatitis in HCC patients, and the post-TACE reactivation of HBV is associated with poor outcome [53,54]. Prophylactic antiviral therapy can significantly reduce the risk of post-TACE reactivation of hepatitis B [54,55].

Tyrosine kinase inhibitors including sorafenib and lenvatinib are used for the treatment of advanced HCC. The reactivation of hepatitis B has been reported in HBV patients undergoing treatment with sorafenib without receiving antiviral therapy. The prophylactic use of antiviral therapy in patients with HBV-related HCC who are candidates for receiving sorafenib is reported to be associated with improved overall survival and is determined as a cost-effective approach [56,57].

Immunotherapy with immune checkpoint inhibitors as inhibitors of programmed cell death protein 1 (PD-1) and programmed cell death ligand 1 (PD-L1) is increasingly used for the treatment of advanced HCC. The reactivation of Hepatitis B has been reported in patients with HBV infection treated with immunotherapy for HCC who did not receive prophylactic antiviral therapy [58]. Although the risk of reactivation does not seem to be high, possibly due to the anti-HBV activity of PD-1 and PD-L1 inhibitors, antiviral treatment should be considered in patients who are positive for HBsAg. The role of prophylactic antiviral therapy in patients with a resolved hepatitis B and positive for anti-HBc but negative for HBsAg is not clear and needs further future studies [55].

## 10. Prognosis of HCC in Hepatitis B

Long-term HBV treatment in HCC has been shown to improve overall survival. A meta-analysis by Yuan et al. demonstrated that overall survival was higher in those who received antiviral treatment with NA, by 11% at 1 year, 28% at 3 years, and 40% at 5 years [59] compared to those who were untreated. Similarly, Zhang et al. showed that antiviral therapy improved the 1-, 3-, and 5-year recurrence-free survival and decreased the mortality rate when compared to untreated controls [60]. In a randomized control trial, post-HCC resection antiviral treatment with lamivudine, adefovir dipivoxil plus lamivudine, or entecavir for those with antiviral resistance was shown to be associated with a reduction in tumor recurrence and decreased HCC-related mortality [61].

In summary, NA therapy post-HCC treatment improves overall survival, and data suggest that NA should be started as soon as possible in those with a diagnosis of HCC and that it should be continued indefinitely.

## 11. Conclusions and Remarks

The prevalence of HBV infection varies across geographic regions: it is highly prevalent in Asia and sub-Saharan Africa, while it has a lower prevalence in parts of Europe, Japan, and North America. It is critical to screen high-risk populations such as individuals with HIV or HCV coinfection, pregnant persons, and people with comorbidities such as diabetes mellitus and metabolic syndrome.

The genetic variability of HBV contributes to disease progression and can influence treatment responses. HBV induces HCC through a variety of mechanisms such as genomic instability, the activation of cancer-promoting pathways (e.g., Wnt/beta-Catenin and PI3K/Akt), and epigenetic modifications. Furthermore, HBV-HDV coinfection drives liver injury and fibrosis and is associated with more rapid progression to end-stage liver disease and HCC.

The treatment strategies for chronic HBV infection and HCC have evolved significantly and continue to advance rapidly. First-line antiviral therapies, such as entecavir and tenofovir, have proven highly effective in reducing the risk of liver complications, including HCC. Long-term NA therapy has been shown to prevent disease progression and reduce the recurrence of HCC. Additionally, systemic therapies for HCC have improved overall survival in patients with inoperable disease. Future studies will need to focus on the role of novel biomarkers to improve HCC detection at an early stage and the development of novel antiviral and immune modulatory therapy that can further reduce mortality associated with HBV-related HCC.

## Figures and Tables

**Table 1:** Description of different mechanisms of HCC induced by hepatitis B.

Mechanism	Description
HBV Gene Integration and Genomic Instability [21]	HBV integrates into the host genome, causing mutations and instability, potentially leading to HCC.
Activation of Cancer-Promoting Pathways [37]	The activation of signaling pathways like Wnt/beta-catenin and PI3K/Akt drive cell proliferation and tumor survival.
Epigenetics [30]	Epigenetic modifications, including methylation and histone changes, contribute to liver cancer progression.
Exosomes and Autophagy [23]	Exosomal signaling and autophagy contribute to immune suppression and metabolic changes, promoting HCC.
Immune Suppression [22]	HBV-induced immune suppression helps tumor cells evade immune responses, promoting cancer progression. This is mainly through CD8+ T cells and tumor-associated macrophages.
ORF X [24]	This ORF encodes the HBx protein, a key factor in oncogenesis and liver cancer development.
Oxidative Damage and Tumor Survival [23]	HBV proteins (HBx, HBs, and HBc) cause oxidative stress, promoting liver fibrosis and cancer progression.
Pro-Oncogenic MAPK14 Activation [23,28]	MAPK14 activation contributes to HBV replication and the survival of tumor cells in liver cancer.

HBV, hepatitis B virus; HBc, hepatitis B core; HBs, hepatitis B surface; HBx, hepatitis B X; HCC, hepatocellular carcinoma; ORF, open reading frame.

**Table 2:** Treatment modalities for hepatocellular carcinoma.

Surgical Treatment
Liver transplantation
**Local ablative therapy** Thermal ablation therapies Radiation segmentectomy External beam radiation therapy
**Transarterial therapy** Transarterial chemoembolization Transarterial radioembolization
**Systemic therapy** Antiangiogenic targeted therapies Immunotherapy

## Data Availability

No new data were created or analyzed in this study.
